# Comparison of the In Vitro Iron Bioavailability of Tempeh Made with *Tenebrio molitor* to Beef and Plant-Based Meat Alternatives

**DOI:** 10.3390/nu16162756

**Published:** 2024-08-18

**Authors:** John W. Wilson, Tyler W. Thompson, Yuren Wei, Jacqueline M. Chaparro, Valerie J. Stull, Mahesh N. Nair, Tiffany L. Weir

**Affiliations:** 1 Department of Food Science and Human Nutrition, Colorado State University, Fort Collins, CO 80523, USA; 2 Department of Animal Sciences, Colorado State University, Fort Collins, CO 80523, USA; 3 Analytical Resources Core, Bioanalysis and Omics (ARC-BIO), Colorado State University, Fort Collins, CO 80523, USA; 4 Center for Sustainability and the Global Environment, University of Wisconsin–Madison, Madison, WI 53726, USA

**Keywords:** edible insects, ferritin, inductively coupled plasma-mass spectrometry, iron bioavailability, plant-based meat alternative, mealworm, *Tenebrio molitor*

## Abstract

Iron is an essential mineral that supports biological functions like growth, oxygen transport, cellular function, and hormone synthesis. Insufficient dietary iron can lead to anemia and cause fatigue, cognitive impairment, and poor immune function. Animal-based foods provide heme iron, which is more bioavailable to humans, while plant-based foods typically contain less bioavailable non-heme iron. Edible insects vary in their iron content and may have heme or non-heme forms, depending on their diet. Edible insects have been proposed as a protein source that could address issues of food insecurity and malnutrition in low resource contexts; therefore, it is important to understand the bioavailability of iron from insect-based foods. In this study, we used Inductively Coupled Plasma and Mass Spectrometry (IPC-MS) and Caco-2 cell culture models to compare the soluble and bioavailable iron among five different lab-produced tempeh formulations featuring *Tenebrio molitor* (mealworm) with their non-fermented raw ingredient combinations. Finally, we compared the iron bioavailability of a mealworm tempeh with two sources of conventional beef (ground beef and sirloin steaks) and two commercially available plant-based meat alternatives. The results show that while plant-based meat alternatives had higher amounts of soluble iron, particularly in the Beyond Burger samples, the fermented mealworm-based tempeh had greater amounts of bioavailable iron than the other samples within the set. While all the samples presented varying degrees of iron bioavailability, all products within the sample set would be considered good sources of dietary iron.

## 1. Introduction

Iron is an essential mineral that is found naturally in a variety of foods. It is a constituent part of oxygen transport molecules, like hemoglobin and myoglobin, and is required for muscle metabolism and the maintenance of healthy connective tissue. Iron supports a variety of essential biological functions including physical growth, neurological development, cellular function, and hormone synthesis [1]. Insufficient iron uptake can lead to a diminished capacity to transport oxygen to the tissues and organs of the body, resulting in a physiological condition known as anemia. Anemia caused by iron deficiency can significantly affect cognitive function, physical activity, and immune function. It has also been associated with complications during pregnancy and maternal mortality, as well as a variety of other health issues [2,3]. While dietary requirements for iron vary depending on age, biological sex, and pregnancy status (Table 1), the FDA requires food labels to list their percent daily value iron content based on an 18 mg/day requirement. For a food to be considered a good source of iron, it must contain at least 20%, or 3.6 mg, of the daily value of iron [3]. Humans, on average, consume between 10–15 mg of dietary iron per day [3]. However, iron deficiency is a prevalent form of malnutrition globally, impacting approximately 30% of the population including 29% of non-pregnant women, 38% of pregnant women, and 43% of children worldwide [4] (WHO, 2024). Furthermore, the source of iron and other components present in a food can impact iron bioavailability. 

Dietary iron is accessed in two forms: heme and non-heme [1]. Heme iron, a ferrous (Fe^2+^) iron chelated into a porphyrin ring structure, is typically found in the hemoglobin and myoglobin of animal proteins like red meat, poultry, and seafood, and is more bioavailable than non-heme iron [6], largely due to the different pathways the types of iron use to enter enterocytes. Non-heme iron accessed from the diet can be ferrous (Fe^2+^) or ferric (Fe^3+^), but ferric iron must be converted to ferrous by duodenal cytochrome B (DCYTB) before it can be transported into the enterocyte through the Divalent Metal Transporter 1 (DTM1) [7]. Heme iron can bypass this and enter the enterocyte through heme transporters (HCP1) [8]. As a result, it is estimated that ~10–20% of consumed heme iron is absorbed while the absorption of non-heme iron is much more variable [9]. Non-heme iron is found in plants and iron-fortified foods like leafy green vegetables, nuts, fortified grains, and legumes. The absorption of iron sourced from dark leafy greens is between 7–9%, while we only absorb roughly 4% of the iron contained in grains and 2% of iron found in legumes [9]. 

Because of the disparity in bioavailability, heme iron is a more significant source of dietary iron than non-heme, plant-based sources [6]. However, interaction between food components and food preparation methods can directly influence the bioavailability of non-heme iron. For example, Vitamin C may improve the absorption of non-heme iron [10]. In addition, lactic fermentation has been shown to improve iron bioavailability in fermented foods like maize, soybeans, and sorghum [11], whereas the solid-state fermentation of black-eyed peas improved their iron bioavailability [12]. On the other hand, chelating agents found in plant foods, such as phytates and polyphenols, can reduce the bioavailability of non-heme iron [10].

Understanding iron bioavailability from different food sources is important in contexts where meat is rarely consumed or intentionally omitted from the diet. For example, in many low-resource contexts iron deficiency is prevalent because dietary animal protein is scarce [13]. In addition, diets that intentionally omit animal products for reasons of religious observance or due to human and planetary health concerns are common and can also lead to iron deficiencies. Edible insects may serve as an alternative protein source in these situations, although previous studies are equivocal regarding their ability to supply bioavailable dietary iron because chitin present in insects can bind to iron, reducing its bioavailability [14,15]. In a recent study comparing the mineral content of grasshoppers, crickets, mealworms, and buffalo worms to sirloin beef, researchers observed significantly higher amounts of soluble iron in the insect samples, but the bioavailability of that iron varied among species. The researchers attributed this to the lack of hemoglobin and myoglobin in most insect species [15]. Further research is needed to assess iron bioavailability from insect-based foods and compare them with animal protein as well as with plant-based meat alternative products, whose global sales were roughly USD 29 billion in 2020 and are expected to reach USD 125 billion by 2030 [16].

In this study, we examined the iron bioavailability of lab-made tempeh formulations produced with varying ratios of soybean and *Tenebrio molitor* (mealworm) larvae and pupae. Although mealworms are one of the few edible insects that are commercially farmed for human food, little is known about levels of soluble iron or its bioavailability, particularly whether differences exist between larval and pupal stages and whether solid-state fermentation impacts these factors. Additionally, because chitin is formed by solid-state fermentation with *Rhizopus oligosporus*, and fermentation has been shown to impact iron bioavailability, we compared the tempeh products to their raw ingredients. Finally, we compared the nutritional content of the insect tempeh preparations with two conventional beef products and two popular plant-based meat alternatives. Specifically, we looked at the differences in soluble iron (based on simulated gastrointestinal digestion) and in vitro iron bioavailability among fermented and non-fermented tempeh preparations with edible insects. Additionally, we compared an insect-based formulation to two beef products and three plant-based meat alternatives (Beyond Burger, Impossible Burger, and lab-produced soy tempeh) using a well-established Caco-2 cell culture model.

Here, we address differences in soluble iron and in vitro iron bioavailability among fermented and non-fermented tempeh preparations containing edible insects, as well as comparing an insect-based formulation to two beef products, and three plant-based meat alternatives (Beyond Burger, Impossible Burger, and lab-produced soy tempeh) using a well-established Caco-2 cell culture model. Based on the current literature, we hypothesized that the presence of animal protein in the insect-based tempeh and the reduction in chelating agents due to *Rhizopus oligosporus* fermentation would increase the bioavailability of the iron contained within the tempeh products, rendering them as rich a source of iron as the beef and potentially superior to plant-based meat alternatives.

## 2. Materials and Methods

### 2.1. Mealworm Sourcing

All mealworms were procured from a commercial producer (Rainbow Mealworms, Compton, CA, USA) and fed a diet of wheat bran, lyophilized brewer’s yeast, and carrots for a minimum of five days prior to processing. The mealworms were then euthanized with liquid nitrogen and stored at −20 °C until use. Pupae were acquired by allowing the mealworms to enter the pupal stage of their development, separating them from the remaining stock, and euthanizing them with liquid nitrogen before storing at −20 °C until use.

### 2.2. Tempeh Production

Five different variations of tempeh were produced in the lab for this project: 100% soy, 100% mealworm (*Tenebrio molitor* larvae), 100% pupae (*Tenebrio molitor* pupae), 50/50 mealworm/soy by weight, and 50/50 pupae/soy by weight. The 100% soy tempeh was made by soaking 400 g of dry soybeans in deionized (DI) water for 24 h prior to processing. After soaking, the soybeans were boiled for 90 min, drained, and cooled at room temperature for 20 min. Once cool, the soybeans were manually broken apart to separate the husk from the inner flesh and 2 mL of distilled white vinegar was added. The soybeans and vinegar were mixed for 60 s followed by inoculation with 20 g of *Rhizopus oligosporus* spores mixed with rice flour and soy flour (Wira, Pemona, CA, USA), and the substrate was mixed for an additional 60 s. 

The 100% mealworm larvae and 100% pupae tempeh preparations were produced from 400 g each of frozen mealworm larvae or pupae, which were blanched in boiling water for 60 s. After blanching, the mealworm larvae and pupae were allowed to cool for 20 min. The procedure then followed the same process as soybean tempeh with the addition of 2 mL white vinegar, followed by inoculation with 20 g of *Rhizopus oligosporus* spores, rice flour, and soy flour. 

The 50/50 mealworm (larvae and pupae) and soy mixtures were prepared as above except 200 g of either mealworm larvae or pupae was mixed with 200 g of soaked and dehulled soybeans prior to acidification and inoculation. Each mixture was then placed into six quart-sized plastic bags, each with five rows of six perforations spaced 1 cm apart. Excess air was removed from each bag, and all samples were placed in an incubator at 30 °C and 80% relative humidity (RH) for 24 h. Non-fermented controls were produced by preparing and mixing raw ingredients in the portions specified above, but the acidification and inoculation steps were omitted.

### 2.3. Sample Cooking and Homogenization

All ground and whole meat and plant-based alternative products were collected from various grocery stores in Fort Collins, CO, to ensure different lot production numbers of ground beef (80% lean, 20% fat), sirloin steaks, Beyond Burger, and Impossible Burger. All products were cooked in a Rational oven (Model No. SCC WE 61; RATIONAL AG, Landsberg am Lech, Germany) on the dry heat setting at 95 °C with ground products being cooked to an internal temperature of 71 °C, and 65 °C for whole-muscle cuts. Following cooking, products were chilled in a walk-in cooler for 30 min before being homogenized. The homogenization process consisted of freezing products in liquid nitrogen and immediately homogenizing them in a Robo Coupe BLITZER 6V (Robot Coupe USA Inc., Ridgeland, MS, USA) blender. The samples were then sealed in vacuum bags and frozen at −20 °C until further processing.

All tempeh products were cut into 25 mm cubes and cooked on each side in a dry pan set to medium-high to an internal temperature of 37 °C. The tempeh control products consisting of non-fermented raw ingredients were heated in a dry pan at medium-high heat for five minutes. Following cooking, products were chilled in a walk-in cooler for 30 min before being homogenized. The homogenization process consisted of freezing products in liquid nitrogen and immediately homogenizing them in a Cuisinart 120V spice grinder model DCG-12BC. The samples were then sealed in vacuum bags and frozen at −20 °C until further processing.

### 2.4. Gastrointestinal Digestion

One gram of each sample was added to 10 mL of isotonic saline solution (140 nM NaCl and 5 mM KCl) and vortexed for 10 s to homogenize. The pH of the solution was adjusted to 2.0 using HCl (1 M). Then, 0.5 mL of 16 mg/mL pepsin (Sigma, St. Louis, MO, USA) was added, and the samples were incubated at 37 °C for 75 min. After incubation, peptic digestion was terminated by increasing the pH to 5.5 with NaHCO_3_ (1M). Next, 2.5 mL of 7 mg/mL bile–pancreatin (Sigma, St. Louis, MO, USA) was added to the samples and the pH was increased to 7.0 with NaHCO_3_ (1M) to simulate digestion. The solution was increased to 16 mL using isotonic saline solution and the samples were incubated at 37 °C for 120 min, after which they were centrifuged at 3000× *g* for five minutes. The supernatant was then collected for microwave digestion.

### 2.5. Microwave Digestion

**Figure 1:**
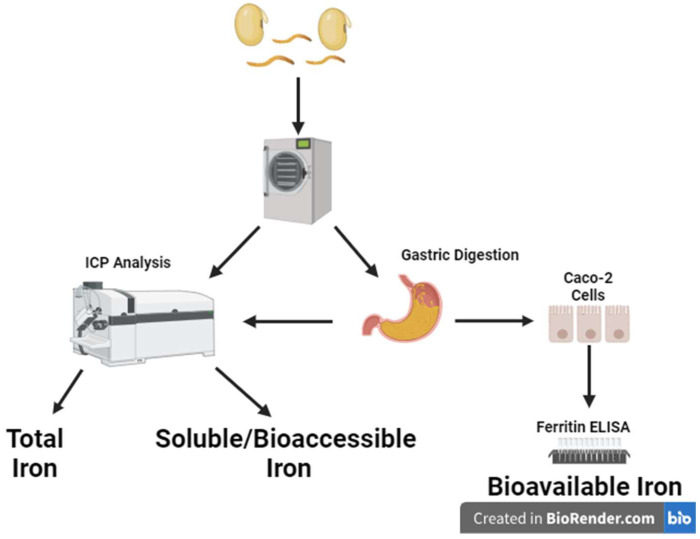
A description of the methodology of this project. Substrates were cooked, freeze-dried, and ground prior to further processing. Total iron samples were processed via microwave digestion and analyzed via ICP-MS. Samples for soluble iron were chemically digested prior to analysis via ICP-MS. Iron bioavailability was determined by adding simulated gastrointestinal digested samples to Caco-2 cells which were then analyzed by ELISA assay for total ferritin.

Microwave digestion was conducted utilizing a Titan MPS microwave digester (PerkinElmer, Waltham, MA, USA) prior to ICP analysis (Figure 1). To determine the total ferritin content of the gastrointestinal digests, samples were prepared by adding either 1 g of lyophilized, powdered sample (total iron) or 1 mL of digested supernatant (soluble iron) to the microwave vessel with 9 mL of nitric acid. The samples were allowed to sit for 15 min to allow for an initial reaction. Then, the samples were placed in the microwave for 1 h. Once cooled, the samples were decanted into 50 mL conical tubes and diluted to 20 mL with milliQ water. One mL of this was added to a 15 mL conical tube and further diluted to 15 mL with milliQ water prior to ICP analysis. 

### 2.6. Inductively Coupled Plasma and Mass Spectrometry (ICP-MS) Analysis

Microwave digested samples of chemical digesta were analyzed by ICP-MS to determine total micronutrient profiles (whole-product samples) and soluble iron (sample digesta) (Figure 1). Elemental iron concentration was measured using a NexION 350D mass spectrometer (PerkinElmer, Waltham, MA, USA) connected to a PFA-ST nebulizer (Thomas Scientific, Swedesboro, NJ, USA) and a Peltier controlled quartz cyclonic spray chamber (PerkinElmer, Waltham, MA, USA) set to 4 °C. Elemental concentrations Fe were measured using a NexION 350D mass spectrometer (PerkinElmer; Waltham, MA, USA) connected to a Type A quartz MEINHARD^®^ concentric nebulizer and a quartz cyclonic spray chamber (Meinhard, Golden, CO, USA). Samples were introduced using a SC-2DX autosampler (ESI). Elemental Fe was measured in Dynamic Reaction Cell (DRC) mode using ammonia as the reactive gas. Before analysis, the torch alignment, nebulizer gas flow, and the Quadrupole Ion Deflector (QID) were optimized for maximum indium signal intensity. A daily performance check was also run which ensured that the instrument was operating properly and minimized oxide and doubly charged species formation by obtaining a CeO^+^:Ce^+^ of <0.025 and a Ba^++^:Ba of <0.03. A calibration curve was obtained by analyzing 7 dilutions of a multi-element stock solution made from a mixture of single-element stock standards (Inorganic Ventures, Christiansburg, VA, USA). To correct for instrument drift, a quality control (QC) solution, which consisted of a pooled digested sample, prepared by mixing 1 mL of each digested individual sample, was run every 10th sample. Calibration was confirmed by a 7-point curve prepared by serial dilution of commercially available single element standard stock solutions. Limits of detection (LOD) and limits of quantification (LOQ) were calculated as 3 times or 10 times the standard deviation of the blank divided by the slope of the calibration curve, respectively, and were subsequently corrected for dilution factor [17,18]. Final concentrations are given in ng/mg of digested sample. Measured calculations below the LOD were assigned as <LOD.

### 2.7. Caco-2 Cell Assay

In vitro iron bioavailability was measured using a Caco-2 cell line ATCC HTB-37, which is an immortalized human colonic cell line that can mimic the function of intestinal enterocytes and is well established as a model for determining iron bioavailability [15,19,20]. Cells between passages 4–6 were grown in tissue culture treated flasks and kept in an incubator at 37 °C and 5% CO_2_ in Dulbecco’s modified Eagle’s medium (DMEM; Hyclone, Logan, UT, USA) supplemented with 10% fetal bovine serum (FBS; Hyclone, Logan, UT, USA), 1% minimum essential media (MEM; Gibco, Billings, MT, USA), and 1% penicillin (Life Tech, Carlsbad, CA, USA). Confluent cells were trypsinized and sub-cultured to 12-well plates, seeded at a density of 2 × 10^5^ cells/mL. Medium was changed every other day for 14 days. DMEM was replaced with MEM 24 h prior to adding heat-inactivated cricket digests. After 24 h, MEM was replenished and cricket digests, normalized to ~100 uM elemental iron per well, were added to cells and incubated for 2 h at 37 °C and 5% CO_2_. After this incubation period, cells were supplemented with an additional 0.5 mL MEM and incubated for another 22 h, after which they were washed with PBS buffer, lysed, and centrifuged for 4 min at 19,000× *g*, and the supernatant was collected for the determination of ferritin levels.

### 2.8. Ferritin Analysis

Sample ferritin content was analyzed utilizing a Human Ferritin ELISA Kit (Sigma-Aldrich Saint Louis, MO, USA) according to manufacturer’s instructions. Thirty ug of supernatant of each sample from the CaCo-2 incubations was added to the 96-well plate and incubated and analyzed on a BioTek Gen5 Microplate reader and Analysis Software. Ferritin levels were normalized to total protein as previously described [21].

### 2.9. Statistical Analysis

All samples were tested with an *n* = 5–6 and outliers were identified and removed using ROUT (Q = 1%). Data were statistically analyzed using GraphPad Prism version 10.1. Data (GraphPad Software Inc., La Jolla, CA, USA) were one-way ANOVA with Tukey’s test for multiple comparisons to identify differences across and among sample groups. When there was unequal variance in the standard deviation between groups, aWelch’s test was applied. Dunnett’s test was used to compare groups to a single control and paired *t*-tests were used to compare between fermented and non-fermented versions of the insect preparations. A *p*-value of <0.05 was considered statistically significant whereas *p*-values between 0.080–0.051 were considered as trending towards significance. 

## 3. Results 

### 3.1. Comparison of Total Iron, Soluble Iron, and Iron Bioavailability of Insect-Based Tempeh Compared to Soybean-Based Controls

**Figure 2:**
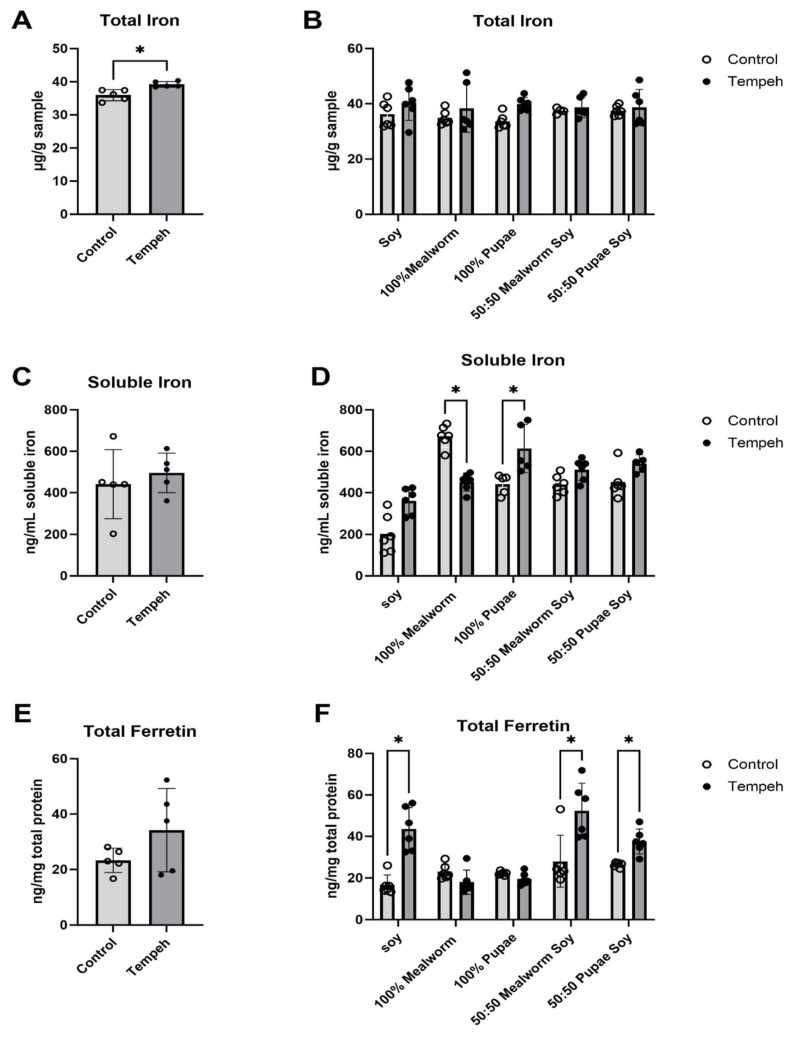
Comparison of total (**A**,**B**), bioaccessible (soluble iron; (**C**,**D**)), and bioavailable (total ferritin; (**E**,**F**)) iron between fermented tempeh and non-fermented raw ingredients (marked control) of different mealworm and soybean preparations. * Highlights treatments with means that are significantly different at *p* < 0.05. Open circles and lighter colored bars represent control preparations while closed circles and darker bars are fermented preparations.

Lyophilized, ground samples or simulated gastrointestinal digests of prepared soy, mealworm larvae, and mealworm pupae formulations were compared for levels of total iron, soluble iron, and bioavailable iron before (control) and after fermentation (tempeh). Tempeh is produced by solid-state fermentation with *Rhizopus oligosporus* mold, and while some fungi can release bound micronutrients through production of phytases that inhibit phytic acid [22,23], they can also produce siderophores that can bind iron, reducing its bioavailability [24,25]. Therefore, we examined how colonization by *Rhizopus* impacts iron status in our tempeh samples. When total elemental iron was examined, there was a small but significant increase after fermentation (Figure 2A; *p* = 0.027). However, when looking individually at control and fermented formulations (Figure 2B), there were no statistically significant changes (*p* > 0.05) in total iron after fermentation. This suggests that fermentation had an overall positive effect on total iron, but there might have been insufficient sample size to detect increases in individual substrate preparations. Because the total iron measurements do not demonstrate bioaccessibility or bioavailability, the increase seen with fermentation may be attributed to the iron content of the fungal mycelium. However, *Rhizopus* does not produce edible fruiting bodies and is not consumed alone as a food source, so there is little information on its specific nutritional properties.

We further examined total soluble (bioaccessible) iron in control versus fermented samples after a simulated gastrointestinal digestion protocol. Unlike total iron, there were no differences in soluble iron between fermented samples and non-fermented controls (Figure 2C; *p* = 0.489). This may be due to the high variability in soluble iron between substrate types. Specifically, there was more soluble iron in 100% mealworm samples than in the 100% soybean controls (*p* < 0.001). When comparing fermented and control pairs within each substrate type, there was significantly more soluble iron in 100% mealworms before fermentation than there was after fermentation (Figure 2D; *p* < 0.001). In contrast, the 100% pupae had significantly less soluble iron before fermentation than after fermentation (adjusted *p* = 0.041). There was also a trend (*p*-values between 0.051–0.08) towards increased soluble iron after fermentation in the 100% soybean (*p* = 0.071) and 50:50 pupae/soybean preparations (*p* = 0.071). Overall, this suggests that the tempeh fermentation process may have had a small effect on total soluble iron. However, the contribution is largely insignificant, probably due to the very short solid-state fermentation times, with our lab-produced samples fermented for only 24 h. This short fermentation may represent a homeostasis between the micronutrients released by fungal enzymes and those taken up and utilized to support rapid mycelial growth. Furthermore, other reports of fermentation-related increases in micronutrients, such as iron, are often attributed to the activity of lactic acid bacteria (LAB) [11]. However, the short fermentation time, neutral pH of the substrate, and aerobic environment limit the presence of LAB on tempeh. 

Finally, we used a Caco-2 cell culture model to explore in vitro iron bioavailability, determined by the amount of ferritin detected in lysed cells after a 24 h incubation with neutralized gastrointestinal digests of each sample. Overall, there was no significant change in iron absorption with fermentation (Figure 2E; *p* = 0.174), although there was a high amount of variability in bioavailable iron between the different substrates. Within substrate comparisons showed that samples containing soy (Figure 2F; *p*-values for 100% soy: *p* < 0.001; 50:50 mealworm/soy: *p* = 0.025; and 50:50 pupae/soy: *p* = 0.005) consistently resulted in increased levels of ferritin after fermentation. When comparing the amount of ferritin across substrates, there was no significant difference between the 100% soy tempeh and the two insect formulations that contained a mixture of insects and soy. However, the 100% mealworm larvae and pupae formulations had significantly less bioavailable iron, indicated by reduced ferritin (adjusted *p*-values for each formulation compared to the soy control: *p* < 0.001). The 50:50 mealworm/soy formula had superior sensory qualities compared to the 50:50 pupae/soy formulation [26], and mealworm larvae are also comparatively easier to raise and commercially source. Therefore, the 50:50 mealworm/soy preparation was considered the most viable preparation for further testing to determine suitability as a product for widespread consumption. 

### 3.2. Comparison of Soluble and Bioavailable Iron from Meat with Plant- or Insect-Based Meat Alternatives

We compared the iron bioavailability of this insect-based preparation and the traditional soybean tempeh to meat (sirloin and ground beef) and plant-based meat alternatives.

**Figure 3:**
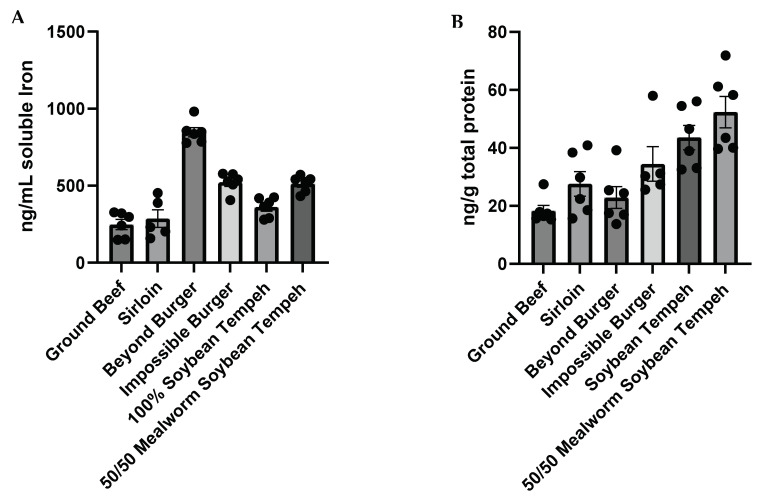
Total bioaccessible/soluble (**A**) and bioavailable (**B**) iron in different meat and plant- or insect-based meat alternatives.

After simulated gastrointestinal digestion, the Beyond Burger samples had higher (*p* < 0.05) total soluble iron compared to all other samples (Figure 3A; Table 2). This was followed by the Impossible Burger and 50:50 mealworm/soy tempeh, which were similar (*p* > 0.05) in bioaccessible iron to each other but had higher levels (*p* < 0.05) than those observed in the two beef and 100% soybean samples. 

Similar to the results reported for the insect-based samples, the amount of total soluble iron was not a good predictor of iron bioavailability. The 50:50 mealworm/soy tempeh was associated with the highest (*p* < 0.05) ferritin levels, which were significantly greater than those reported for Beyond Burger, sirloin, or ground beef samples (Figure 3B, Table 3). Likewise, the 100% soy tempeh preparation resulted in significantly higher levels of ferritin than either the Beyond Burger or the ground beef. There were no other significant differences observed.

## 4. Discussion

This study is the first to compare iron bioavailability in foods after undergoing a solid-state fermentation process and one of the few studies comparing conventional meat and plant-based meat alternatives. One of the most significant observations in this study was that neither total iron nor soluble iron was predictive of iron bioavailability in conventional beef, plant-based meat alternatives, or tempeh preparations. For example, the plant-based meat alternative samples had higher amounts of soluble iron when compared to the other samples in the study; however, these increased amounts of iron did not result in greater iron bioavailability. Iron bioavailability is influenced by both the iron inhibitors (antinutrients and other chelators) and enhancers (heme form of iron, vitamin C) found in the food matrix. Anti-nutrients such as phytic acid, tannin, calcium, and dietary fiber are the main inhibitors of iron absorption [27]. Although plant-based foods are rich in iron, the non-heme form along with the abovementioned factors often limit iron absorption and therefore bioavailability. However, that is contradictory to what we report in this study, where the soybean-containing tempeh products demonstrated greater iron bioavailability than the insect-only preparations or conventional meat products. 

When comparing the lab-produced tempeh samples, the fermentation process was an important factor, as there was an overall effect of fermentation on the iron bioavailability of the soybean-containing fermented preparations (Figure 2E). Soybeans are a rich source of iron, but phytates and polyphenols within the soybeans may inhibit the solubility and bioavailability of the iron. When compared against the soybean control, the soybean tempeh had significantly more iron absorbed. This agrees with previous research indicating that fermentation by *Rhizopus oligosporus* may reduce the chelating agents in soybeans, allowing the iron to be more bioavailable [28,29]. A recent Swedish study looking at potential iron and zinc bioavailability from plant-based meat alternatives showed that tempeh had low phytate content (24 mg/100 g) and high iron (2 mg/100 g), estimating that it might be a good non-animal source of bioavailable iron [30]. Our results confirm these data by suggesting that soybean-containing tempeh products are a good source of bioavailable dietary iron.

While the amount of bioavailable iron detected in the soy control was also significantly less than the 50/50 mealworm/soybean tempeh and the 50/50 pupae/soy tempeh, the soy tempeh had significantly more iron absorption than the mealworm control, pupae control, and the 50/50 pupae/soy control, suggesting that the addition of insect protein alone is not enough to increase the amount of iron absorbed from the sample. Insects contain a small amount of heme iron, but the majority is a protein-bound form of iron called entoferritin [31]. Entoferritin is highly soluble and may have similar bioavailability to iron from other vertebrates, but this has not been adequately explored in relevant models. There is also some evidence that the presence of animal protein in insects improves the bioavailability of non-heme forms of iron [32]. Known as the “meat factor”, there is evidence to suggest that peptides containing higher levels of cysteine can facilitate the absorption of non-heme iron [10]. If this is the case, we would then expect to see similar iron absorption from food sources containing insect-based protein as we see with conventional meat. We found that the 100% insect-containing preparations had similar levels to the conventional meats that we tested (~20 ng/g protein). These concur with previous reports that buffalo worm and mealworm have similar levels of bioavailable iron in a Caco-2 model as sirloin beef [15]. Therefore, it is likely that the entoferritin and the “meat factor” do contribute to iron absorption. 

The other plant-based products, Impossible Burger and Beyond Burger, also did not significantly differ from conventional meat in terms of iron bioavailability. Plant-based meat products are typically formulated to contain similar levels of iron as their conventional meat counterparts; however, the lack of heme iron and the presence of anti-nutrients like oxalates, phytates, and tannins found in plant tissues often reduce iron absorption [10]. To counter these effects, the Beyond Burger is manufactured to include 28 mg of vitamin C [33] and the Impossible Burger utilizes a soy-based leghemoglobin, a plant-based heme containing protein found in the roots of nitrogen-fixing plants, to improve iron bioavailability [34]. Even with the differences in iron uptake between samples, the evidence suggests that all samples examined in this study can be considered high sources of iron as defined by the FDA. 

Finally, it should be noted that there were limitations to the study conduct and interpretation of data. Although we detected statistically significant differences in soluble and bioavailable iron among samples, many of the 99% confidence intervals were larger than the mean differences. Therefore, these differences should be interpreted with caution and a larger sample size would be needed to verify these results. Additionally, there are several limitations of the Caco-2 cell model used to determine iron bioavailability. A critical review by Sandburg [27] highlighted incongruence among studies correlating ferritin formation in Caco-2 cells with the consumption of isotopically labeled foods for estimating iron bioavailability in humans. Additionally, ferritin formation in Caco-2 cells only represents the apical entry of iron into enterocytes, but not its basolateral release into the circulation. Although these aspects are important to acknowledge, the purpose of our study was to compare bioavailable iron among different food products rather than to establish their absolute contribution to iron status in humans. For this purpose, the Caco-2 cell model is an appropriate, high-throughput option [19,27]. 

## 5. Conclusions

The perception that plant-based meat alternatives are healthier than conventional animal protein is driving the growing popularity of these products. The value of this market is expected to grow to USD 30.92 billion by the year 2026 [35]. While plant-based startups like Beyond Meats and Impossible Foods have been leading the industry in the development of plant-based meat alternatives, conventional meat producers including Cargill, JBS, and Tyson are also developing their own products to compete for a share of the market. These products are being formulated to reflect the nutrient composition of conventional meat products, especially in the areas of protein and iron content [10]. The insect-based products evaluated in this study may exist in a space between conventional meat products and plant-based meat alternatives and could serve to address the nutritional needs of a growing population, particularly in lower resource contexts where meat is scarce and insect consumption is culturally accepted. In addition, introducing insects into familiar formats, such as tempeh, could broaden their acceptability in Western cultures that do not have a tradition of insect consumption. Therefore, it is important to understand the true nutritional impact of these meat alternative products.

## Figures and Tables

**Table 1:** Iron requirements in humans across the lifespan [5] (National Institutes of Health: Office of Dietary Supplements, 2023).

Life Stage	Iron RDA
Birth to 6 months	0.27 mg/day
Children 6 months to 13 years	7–11 mg/day
Teenage boys 14–18 years	11 mg/day
Teenage girls 14–18	15 mg/day
Adult men 19–50	8 mg/day
Adult women 19–50	18 mg/day
Adults 51 years and older	8 mg/day
Pregnant women	27 mg/day
Breastfeeding women	10 mg/day

**Table 2:** Comparison of total soluble iron (after simulated digestion) in beef products and plant- and insect-based beef alternatives. One-way ANOVA with Tukey’s post hoc adjusted for multiple comparisons. Differences with *p* < 0.05 were considered statistically significant.

Sample Comparison	Mean Diff.	99% CI	Adjusted *p*
Sirloin vs. Ground Beef	38.87	−138 to 216	0.962
Beyond Burger vs. Ground Beef	599.50	431 to 768	<0.0001
Impossible Burger vs. Ground Beef	274.70	106 to 443	<0.0001
50/50 Mealworm/Soy Tempeh vs. Ground Beef	264.50	96 to 433	<0.0001
100% Soy Tempeh vs. Ground Beef	113.30	−55 to 282	0.157
Beyond Burger vs. Sirloin	560.60	384 to 737	<0.0001
Impossible Burger vs. Sirloin	235.80	59 to 413	0.000
50/50 Mealworm/Soy Tempeh vs. Sirloin	225.60	49 to 402	0.001
100% Soy Tempeh vs. Sirloin	74.47	−102 to 251	0.626
Impossible Burger vs. Beyond Burger	−324.80	−493 to −156	<0.0001
50/50 Mealworm/Soy Tempeh vs. Beyond Burger	−335.00	−503 to −167	<0.0001
100% Soybean Tempeh vs. Beyond Burger	−486.20	−655 to −318	<0.0001
50/50 Mealworm/Soy Tempeh vs. Impossible Burger	−10.17	−179 to 158	>0.9999
100% Soy Tempeh vs. Impossible Burger	−161.30	−330 to 7	0.015
100% Soy Tempeh vs. 50/50 Mealworm/Soy Tempeh	−151.20	−320 to 17	0.026

**Table 3:** Comparison of total bioavailable iron (expressed as ng ferritin/mg total protein) in beef products and plant- and insect-based beef alternatives. One-way ANOVA with Tukey’s post hoc adjusted for multiple comparisons. Differences with *p* < 0.05 were considered statistically significant.

Sample Comparison	Mean Diff.	99% CI	Adjusted *p*
Sirloin vs. Ground Beef	9.25	−13 to 32	0.653
Beyond Burger vs. Ground Beef	4.53	−18 to 27	0.974
Impossible Burger vs. Ground Beef	16.12	−8 to 40	0.148
Soy Tempeh vs. Ground Beef	25.23	35 to 48	0.003
50/50 Mealworm/Soy Tempeh vs. Ground Beef	34.03	11 to 57	<0.0001
Beyond Burger vs. Sirloin	−4.72	−27 to 18	0.969
Impossible Burger vs. Sirloin	6.87	−17 to 31	0.886
Soy Tempeh vs. Sirloin	15.98	−7 to 39	0.122
50/50 Mealworm/Soy Tempeh vs. Sirloin	24.78	2 to 47	0.004
Impossible Burger vs. Beyond Burger	11.59	−12 to 35	0.470
Soy Tempeh vs. Beyond Burger	20.70	−2 to 43	0.022
50/50 Mealworm/Soy Tempeh vs. Beyond Burger	29.50	7 to 52	0.001
Soy Tempeh vs. Impossible Burger	9.11	−15 to 33	0.709
50/50 Mealworm/Soy Tempeh vs. Impossible Burger	17.91	−6 to 42	0.084
50/50 Mealworm/Soy Tempeh vs. Soy Tempeh	8.80	−14 to 31	0.698

## Data Availability

Raw data files available from the authors upon request.
